# YAP ISGylation increases its stability and promotes its positive regulation on PPP by stimulating 6PGL transcription

**DOI:** 10.1038/s41420-022-00842-8

**Published:** 2022-02-11

**Authors:** Xiangfei Xue, Xiaoting Tian, Congcong Zhang, Yayou Miao, Yikun Wang, Yingxiu Peng, Shiyu Qiu, Hong Wang, Jiangtao Cui, Leiqun Cao, Fenyong Sun, Yongxia Qiao, Xiao Zhang

**Affiliations:** 1 Department of Clinical Laboratory Medicine, Shanghai Tenth People’s Hospital of Tongji University, Shanghai, 200072 China; 2 Shanghai Institute of Thoracic Oncology, Shanghai Chest Hospital, Shanghai Jiao Tong University, Shanghai, 200030 China; 3 School of Medicine, Anhui University of Science and Technology, Huainan, Anhui 232001 China; 4 Department of Pharmacy, Shanghai Municipal Hospital of Traditional Chinese Medicine, Shanghai University of Traditional Chinese Medicine, Shanghai, 200071 China; 5 School of Public Health, Shanghai Jiao Tong University School of Medicine, Shanghai, 200025 China; 6 Department of Thoracic Surgery, Shanghai Chest Hospital, Shanghai Jiao Tong University, Shanghai, 200030 China

**Keywords:** Cancer metabolism, Cancer microenvironment

## Abstract

Yes-associated protein (YAP) activation is crucial for tumor formation and development, and its stability is regulated by ubiquitination. ISGylation is a type of ubiquitination like post-translational modification, whereas whether YAP is ISGylated and how ISGylation influences YAP ubiquitination-related function remains uncovered. In addition, YAP can activate glucose metabolism by activating the hexosamine biosynthesis pathway (HBP) and glycolysis, and generate a large number of intermediates to promote tumor proliferation. However, whether YAP stimulates the pentose phosphate pathway (PPP), another tumor-promoting glucose metabolism pathway, and the relationship between this stimulation and ISGylation needs further investigation. Here, we found that YAP was ISGylated and this ISGylation inhibited YAP ubiquitination, proteasome degradation, interaction with-beta-transducin repeat containing E3 ubiquitin-protein ligase (βTrCP) to promote YAP stability. However, ISGylation-induced pro-YAP effects were abolished by YAP K497R (K, lysine; R, arginine) mutation, suggesting K497 could be the major YAP ISGylation site. In addition, YAP ISGylation promoted cell viability, cell-derived xenograft (CDX) and patient-derived xenograft (PDX) tumor formation. YAP ISGylation also increased downstream genes transcription, including one of the key enzymes of PPP, 6-phosphogluconolactonase (6PGL). Mechanistically, YAP promoted 6PGL transcription by simultaneously recruiting SMAD family member 2 (SMAD2) and TEA domain transcription factor 4 (TEAD4) binding to the 6PGL promoter to activate PPP. In clinical lung adenocarcinoma (LUAD) specimens, we found that YAP ISGylation degree was positively associated with 6PGL mRNA level, especially in high glucose LUAD tissues compared to low glucose LUAD tissues. Collectively, this study suggested that YAP ISGylation is critical for maintaining its stability and further activation of PPP. Targeting ISGylated YAP might be a new choice for hyperglycemia cancer treatment.

## Introduction

Yes-associated protein (YAP) is an effector downstream of the Hippo-YAP signaling pathway [1]. As an intracellular connexin and transcriptional co-activator, YAP regulates transcription and signal transduction of multiple genes by binding and activating transcription factors (i.e., TEADs, SMADs, RUNX family transcription factors (RUNXs), tumor protein 73) [2, 3]. YAP has been identified as an oncogene [4, 5]. The abnormal expression of YAP has been found in a variety of malignant tumors, and transgenic expression of YAP in mouse liver reversibly enlarged livers and eventually led to tumor formation suggesting that YAP has an important role in the occurrence and development of tumors [1, 6, 7]. As for LUAD, YAP is activated, promotes cell proliferation, and assists with tumor formation [8, 9]. High expression of YAP predicts poor prognosis in epidermal growth factor receptor (EGFR)-mutant non-small-cell lung cancer (NSCLC) patients [10]. YAP is also involved in metabolic regulation, such as promoting glucose metabolism, lipogenesis, and glutamine utilizing [11–13]. YAP promotes glucose metabolism by stimulating glycolysis and HBP to further stimulate tumorigenesis [14, 15]. These effects maintain YAP as a key oncogenic factor. However, whether YAP promotes another pro-cancer metabolism, such as another form of glucose metabolism PPP, remains uncovered.

Post-translational modification of protein is one of the main ways to regulate YAP function [16]. It has been found that YAP can undergo post-translational modifications such as phosphorylation, O-GlcNAcylation, ubiquitination, SUMOylation, methylation, acetylation, and so on [15, 17–20]. Under normal circumstances, YAP is phosphorylated by large tumor suppressor kinase (LATS) and binds with 14-3-3 protein in the cytoplasm [20]. During cytoplasmic retention, YAP can be further phosphorylated by casein kinase 1 (CK1), ubiquitinated by several E3 ligases, and further degraded in proteasome [21]. When the upstream Hippo signal regulation is abnormal, YAP is dephosphorylated, enters the nucleus, and acts as a transcriptional co-activator to promote tumorigenesis [15].

ISGylation is a ubiquitination-like post-translational modification that interferon-stimulated gene 15 (ISG15) protein covalently linked to the lysine residue of the target protein under the catalysis of E3 ligase [22]. A series of distinct enzymes are involved in the process of protein ISGylation, including ubiquitin-activating E1 enzyme (ubiquitin-activating enzyme E1-like protein, UBE1L), conjugating E2 enzyme (ubiquitin-conjugating human enzyme 8, UbCH8), and several types of ligating E3 ligases [23]. The influence between ISGylation and ubiquitination is very close. ISGylation may occur at the same K site with ubiquitination or close to the ubiquitinated K site, further affecting protein function by competing with ubiquitination [24]. In addition, following viral infection, neural precursor cell expressed developmentally downregulated protein 4 (NEDD4) is ISGylated and blocks the interaction with the ubiquitin-conjugating enzyme (E2), further inhibiting NEDD4-induced ubiquitination to promote anti-viral immune response [25]. Since the ubiquitination of YAP significantly reduces the stability of YAP [21, 26], whether YAP is ISGylated to compete with ubiquitination and the effect of YAP ISGylation on YAP function deserve further investigation.

Here, we found that YAP was ISGylated and its ISGylation increased its stability by inhibiting ubiquitination and interaction with βTrCP. Furthermore, YAP ISGylation was critical for LUAD malignant phenotypes and downstream PPP activation. Targeting YAP ISGylation might be beneficial for hyperglycemia LUAD treatment.

## Results

### YAP ISGylation increases its stability by inhibiting its ubiquitination

**Fig. 1:**
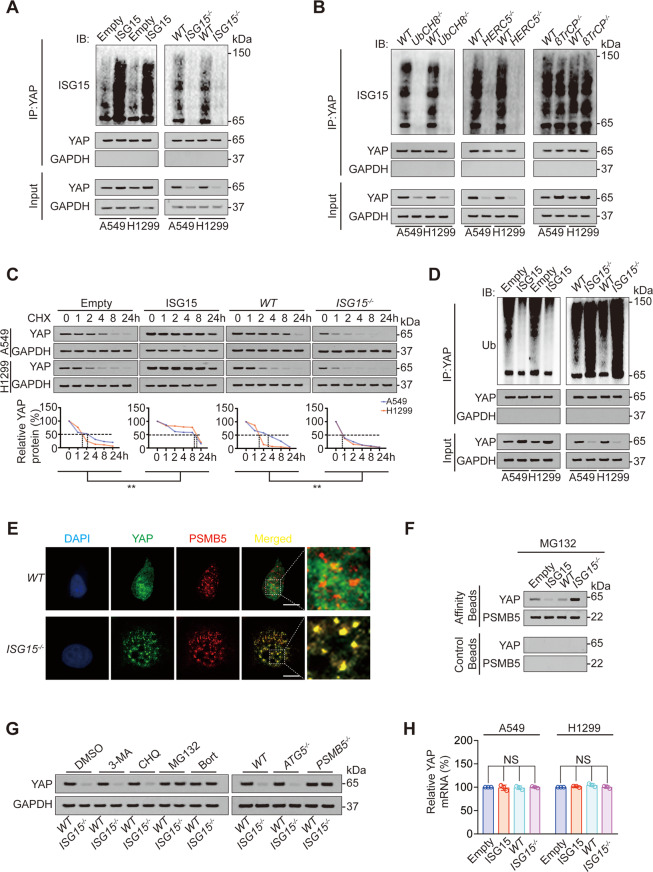
YAP ISGylation inhibits its ubiquitination and increases its stability. **A** Co-IP was performed in control, ISG15 overexpression or knockout A549 and H1299 cells using anti-YAP antibodies. The YAP level in each co-IP sample was adjusted to the same protein content. Indicated proteins were further analyzed by IB. **B** Co-IP was performed in control or UbCH8, HERC5, or βTrCP knockout A549 and H1299 cells using anti-YAP antibodies. The YAP level in each co-IP sample was adjusted to the same protein content. Indicated proteins were further analyzed by IB. **C** CHX (10 μg/ml) chase experiments were performed in control, ISG15 overexpression or knockout A549 and H1299 cells. The relative protein levels of YAP were shown as the ratios between YAP and GAPDH, and the “0 h” points were arbitrarily set to 100%. **D** Co-IP was performed in control, ISG15 overexpression or knockout A549 and H1299 cells using anti-YAP antibodies. The YAP level in each co-IP sample was adjusted to the same protein content. Indicated proteins were further analyzed by IB. **E** Co-localization of YAP and PSMB5 was analyzed in *WT* or *ISG15*^*−/*^^−^ A549 cells before being photographed using a confocal microscope. Scale bar, 20 μm. **F** Association of YAP and PSMB5 analyzed by IB in proteasomes isolated from A549 cells with or without ISG15 overexpression or knockout in the presence of MG132 (8 μM, 24 h). Samples from affinity or control beads were analyzed in parallel. **G** YAP expression was analyzed by IB in *WT* or *ISG15*^*−/−*^ A549 cells with 3-MA, CHQ, MG132, or Bort treatment or with ATG5 or PSMB5 knockout. **H** YAP mRNA level was analyzed in control, ISG15 overexpression or knockout A549, and H1299 cells. The data are shown as the mean ± SD from three biological replicates (including IB). Data in **C** were analyzed using a two-way ANOVA test. Data in **H** were analyzed using a one-way ANOVA test. NS nonsignificant.

Firstly, we investigated whether ISGylation regulated YAP expression. We observed that overexpression of ISG15 and interferon α (IFNα) treatment was significantly induced, whereas knockout of ISG15 significantly inhibited YAP ISGylation and expression in LUAD cell lines A549 and H1299 (Fig. 1A, Fig. S1A). Knockout of UbCH8 (E2 ligase for ISGylation) and HECT and RLD domain containing E3 ubiquitin-protein ligase 5 (HERC5, E3 ligase for ISGylation) also inhibited YAP ISGylation and expression; however, βTrCP (E3 ligase for ubiquitination) knockout had no influence on YAP ISGylation (Fig. 1B, Fig. S1B). The above findings suggested YAP ISGylation elevated YAP expression.

Next, we evaluated how ISGylation regulated YAP expression. Since ISGylation is a type of ubiquitination-like and ubiquitination-associated posttranslational modification [27, 28], we hypothesized that ISGylation upregulated YAP expression via competing for its ubiquitination and enhancing its stability. Using cycloheximide (CHX) chasing experiment, we observed that ISG15 overexpression increased, whereas ISG15 knockout decreased YAP half-life (Fig. 1C). YAP ubiquitination is an important reason for the decreased stability of YAP [21, 29]. As expected, ISG15 overexpression inhibited, whereas ISG15 knockout stimulated YAP ubiquitination (Fig. 1D). In addition, ISG15 knockout promoted the subcellular co-localization between YAP and proteasome 20S subunit beta 5 (PSMB5, one active site of the proteasome, Fig. 1E). In the MG132-treated isolated proteasome, we also found that ISG15 overexpression inhibited, whereas ISG15 knockout promoted the recruitment of YAP to the proteasome (Fig. 1F). The proteasome inhibitor MG132 and bortezomib (Bort) reversed ISG15 knockout-induced YAP degradation, whereas the autophagy inhibitor 3-methyladenine (3-MA) or chloroquine (CQ) had no such function (Fig. 1G). Similarly, PSMB5 knockout reversed ISG15 knockout-induced YAP degradation, whereas knockout of autophagy key factor autophagy-related 5 (ATG5) did not influence such phenomenon (Fig. 1G, Fig. S1C). We also excluded the possibility that ISG15 transcriptionally influenced YAP expression (Fig. 1H). These data suggested that the ISGylation of YAP inhibited its ubiquitination and subsequent proteasome degradation, and upregulated its stability.

### YAP ISGylation inhibits YAP-βTrCP interaction

**Fig. 2:**
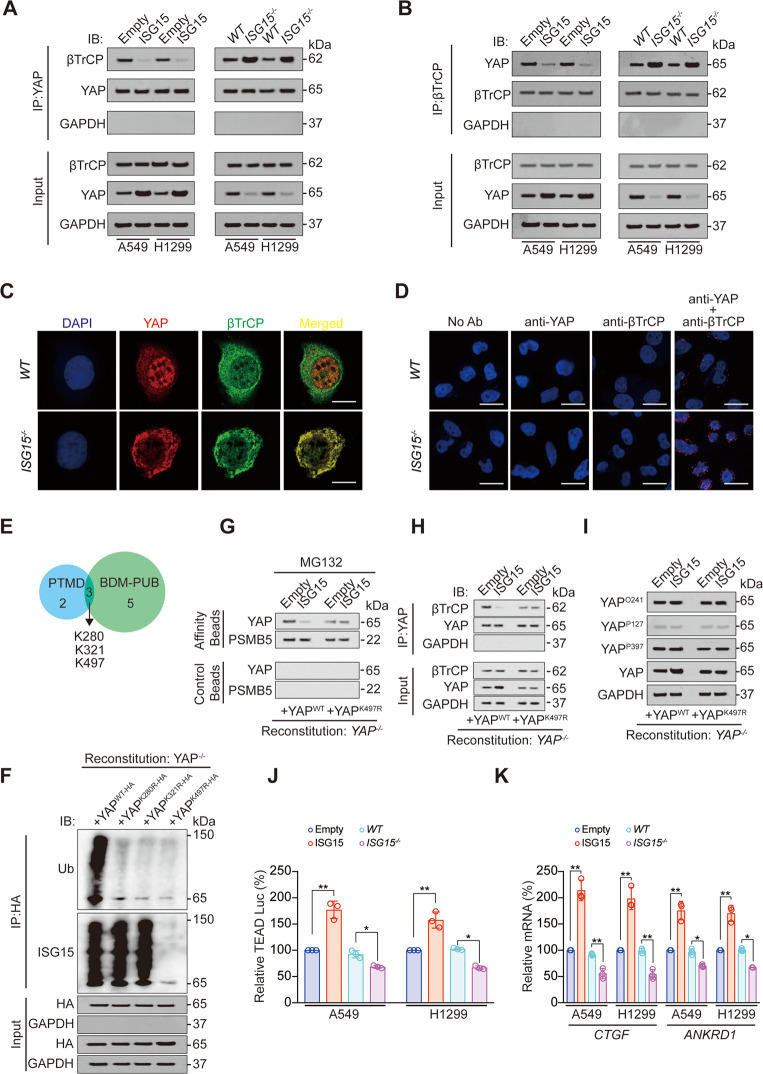
YAP ISGylation inhibits its interaction with βTrCP and promotes downstream transcription. **A**, **B** Co-IP was performed in control, ISG15 overexpression or knockout A549 and H1299 cells using anti-YAP (**A**) or anti-βTrCP (**B**) antibodies. The YAP (**A**) or βTrCP (**B**) level in each co-IP sample was adjusted to the same protein content. Indicated proteins were further analyzed by IB. **C** Co-localization of YAP and βTrCP was analyzed in *WT* or *ISG15*^−*/−*^ A549 cells before being photographed using a confocal microscope. Scale bar, 20 μm. **D** Proximal protein ligation between YAP and βTrCP, as photographed by confocal microscope in *WT* or *ISG15*^*−/−*^A549 cells Scale bar, 50 μm. **E** YAP ubiquitination sites analyzed by PTMD and BDM-PUB databases. **F** Co-IP was performed in *YAP*^−*/*−^ reconstituted A549 cells with YAP^WT-HA^, YAP^K280R-HA^, YAP^K321R-HA^, or YAP^K497R-HA^ expressed. The YAP level in each co-IP sample was adjusted to the same protein content. **G** Association of YAP and PSMB5 analyzed by IB in proteasomes isolated from *YAP*^*−/*^^−^ reconstituted A549 cells with ISG15, YAP^WT-HA^ or YAP^K497R-HA^ expressed in the presence of MG132 (8 μM, 24 h). Samples from affinity or control beads were analyzed in parallel. **H** Co-IP was performed using anti-YAP antibodies in *YAP*^−*/*−^ reconstituted A549 cells with ISG15, YAP^WT-HA^ or YAP^K497R-HA^ expressed. The YAP level in each co-IP sample was adjusted to the same protein content. Indicated proteins were further analyzed by IB. **I** O-GlcNAcylation of YAP at T241 (YAP^O241^), phosphorylation of YAP at S127 (YAP^P127^) and S397 (YAP^P397^) was analyzed in *YAP*^*−/−*^ reconstituted A549 cells with ISG15, YAP^WT-HA^ or YAP^K497R-HA^ expressed by IB. **J**, **K** Relative TEAD Luc (**J**), *CTGF* and *ANKRD1* mRNA level (**K**) were measured using a pUAS-Luc/TEAD-Gal4 system in control, ISG15 overexpression or knockout A549 and H1299 cells. The data are shown as the mean ± SD from three biological replicates (including IB). Data in **J**, **K** were analyzed using a one-way ANOVA test. **P < 0.01, *P < 0.05.

Subsequently, we analyzed whether YAP ISGylation inhibits its interaction with βTrCP. We performed reciprocal co-IP experiments and found that overexpression of ISG15 inhibited, whereas knockout of ISG15 promoted interaction between βTrCP and YAP (Fig. 2A, B). Immunofluorescence and proximity ligation assay (PLA) ligation followed by confocal microscopy analysis revealed that ISG15 knockout stimulated the co-localization between βTrCP and YAP (Fig. 2C, D). These data suggested that YAP ISGylation inhibited YAP-βTrCP interaction.

### YAP is ISGylated at K497

We subsequently explored the potential YAP ISGylation site. Post-translational modifications that are associated with human diseases and prediction of ubiquitination sites with Bayesian discriminant method database were used to predict YAP ubiquitination sites, and we found that K280, K321, and K497 were three YAP ubiquitination sites that both predicted by two databases (Fig. 2E). Since ubiquitination and ISGylation might compete for the same site [24], we performed experiments in YAP^−/−^ reconstituted A549 cells with different YAP mutant proteins expressed, and observed that K280R, K321R, and K497R mutations all significantly declined total YAP ubiquitination, whereas only K497R significantly declined total YAP ISGylation (Fig. 2F), which suggested that K497 might be the major YAP ISGylation site (Fig. 2F). In addition, YAP K497R mutation abolished the inhibitory role of ISG15 for the entry of YAP into proteasome and the combination between YAP and βTrCP (Fig. 2G, H, Fig. S2A, B). The promotive effect of ISG15 on YAP expression was also abolished by K497R mutation (Fig. 2H, I, Fig. S2B, C). We further excluded the possibility that YAP ISGylation regulated YAP O-GlcNAcylation and phosphorylation [15, 20] (Fig. 2I, Fig. S2C). Simultaneously, we observed that ISG15 did not affect the binding of YAP to CK1 and LATS1, whereas increased YAP binding to TEAD4, a key transcription factor that promotes transcription of genes downstream of YAP [30] (Fig. S2D, E). We also found that overexpression of ISG15 stimulated, whereas ISG15 knockdown inhibited TEAD Luc activity, mRNA levels of two YAP downstream genes, *connective tissue growth factor (CTGF),* and *ankyrin repeat domain 1 (ANKRD1)* (Fig. 2J, K) [31]. Therefore, ISGylation regulated YAP independently of the classical upstream Hippo signaling pathway but promoted the downstream transcription of YAP.

### YAP ISGylation is critical for malignant phenotypes

**Fig. 3:**
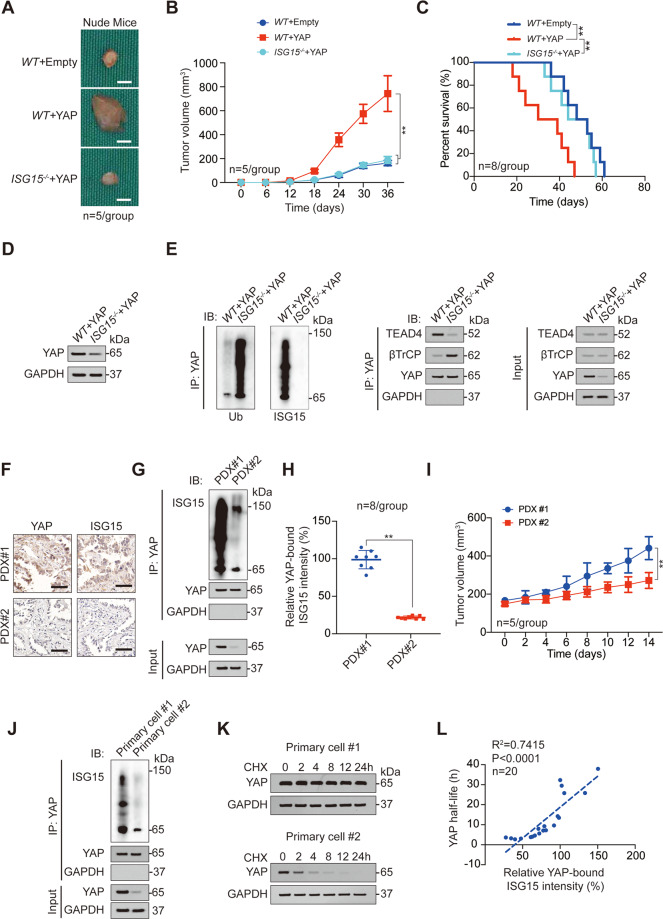
YAP ISGylation is critical for LUAD malignant phenotype. **A**–**C** Representative images (**A**), tumor volume (**B**), and survival analysis (**C**) for xenograft tumor formed by *WT or ISG15*^−^^*/−*^ A549 cells with or without YAP overexpression. **D** YAP expression was analyzed by IB in xenograft tumors formed by *WT or ISG15*^−*/−*^ A549 cells with YAP overexpression. **E** Co-IP experiments analyzing YAP ISGylation, ubiquitination, and interacted proteins in xenograft tumors formed by *WT or ISG15*^*−/*^^−^ A549 cells with YAP overexpression. The YAP level in each co-IP sample was adjusted to the same protein content. **F** YAP and ISG15 expression in PDX#1 and PDX#2 were analyzed by IHC. Scale bar, 200 μm. **G**, **H** YAP ISGylation was analyzed by co-IP experiments and counted in PDX#1 and PDX#2 (*n* = 8). The YAP level in each co-IP sample was adjusted to the same protein content. **I** Tumor volume of PDX#1 or PDX#2 for indicated times. **J** YAP ISGylation was analyzed by co-IP experiments in Primary cell #1 and Primary cell #2. The YAP level in each co-IP sample was adjusted to the same protein content. **K** CHX (10 μg/ml) chase experiments were performed in Primary cell #1 and Primary cell #2. **L** Association between YAP half-life and relative YAP-bound ISG15 intensity was analyzed in 20 primary cell lines. The data are shown as the mean ± SD from three biological replicates (including IB). Data in **B**, **I** were analyzed using a two-way ANOVA test. Data in **C** were analyzed using a log-rank test. Data in **H** were analyzed using a student’s *t* test. Data in **L** were analyzed using a Spearman rank correlation analysis. **P < 0.01.

Given that ISGylation has a great impact on the downstream transcription of YAP, we believed that ISGylation is likely to further affect YAP-related malignant phenotypes. Indeed, we observed that both increased cell viability and decreased apoptosis induced by YAP overexpression could be reversed by ISG15 knockout (Fig. S3A, B). In xenograft models, ISG15 knockdown reversed enlarged tumor size and poor mouse prognosis caused by YAP overexpression (Fig. 3A–C). Mechanistically, ISG15 knockout decreased the expression of YAP, promoted the ubiquitination of YAP, the binding of YAP to βTrCP, and inhibited the binding of YAP to TEAD4 (Fig. 3D, E). We established two series of LUAD PDX models, PDX#1 highly expressed YAP and ISG15, while PDX#2 lowly expressed YAP and ISG15 (Fig. 3F). The ISGylation level of YAP in PDX#1 models was also significantly higher than that in PDX#2 models (Fig. 3G-H). In addition, the transplanted tumor size of the PDX#1 model was significantly larger than that of the PDX#2 model (Fig. 3I), and the prognosis of PDX#1 model mice was worse than that of PDX#2 mice (Fig. S3C). We used the tumor cells constructing the PDX model for primary culture and found that the ISGylation of YAP in Primary cell #1 (used for constructing PDX#1) was significantly higher than that in Primary cell #2 (used for constructing PDX#2), and the half-life of YAP in Primary cell #1 was also significantly higher than that in Primary cell #2 (Fig. 3J, K). Moreover, in the total of 20 primary cell lines constructed from other fresh LUAD tissues, we found that the half-life of YAP was negatively correlated, while the cell viability was positively correlated with YAP ISGylation (Fig. 3L and S3D). These data suggested that YAP ISGylation is critical for malignant phenotypes of LUAD cells.

### YAP stimulates PPP by elevating 6PGL

**Fig. 4:**
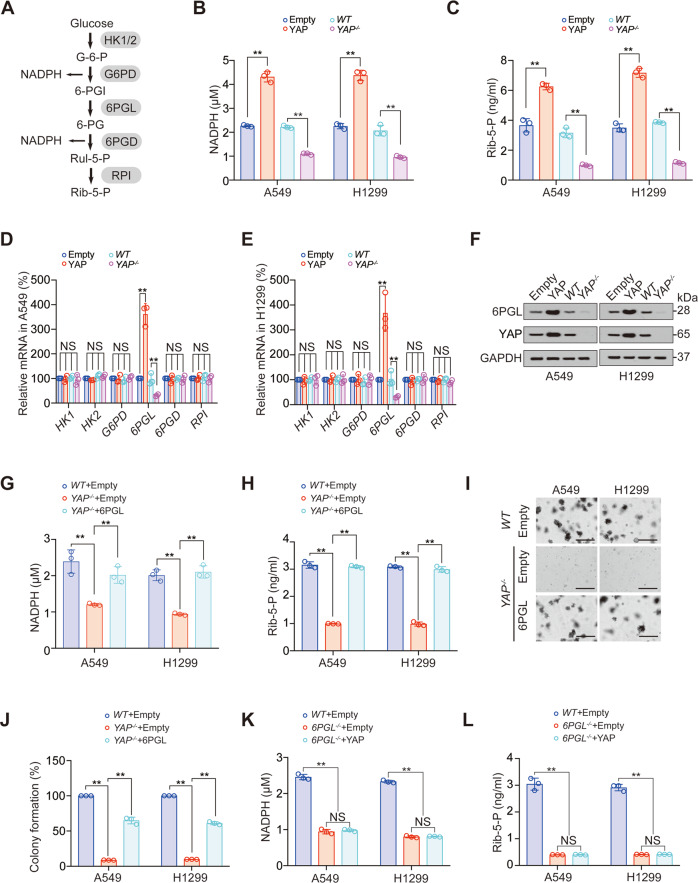
YAP stimulates PPP by activating 6PGL. **A** Schematic presentation of PPP from glucose to Rib-5-P. **B**, **C** NADPH (**B**) and Rib-5-P (**C**) concentration in control, YAP overexpression or knockout A549 and H1299 cells. **D**, **E** Indicated mRNA levels in control, YAP overexpression or knockout A549 (**D**) and H1299 (**E**) cells. **F** 6PGL and YAP protein levels in control, YAP overexpression or knockout A549 and H1299 cells. **G**–**J** NADPH (**G**), Rib-5-P concentration (**H**), and colony formation (**I**, **J**) were measured in *WT* or *YAP*^−*/−*^ A549 and H1299 cells with or without 6PGL overexpression. Scale bar, 200 μm. **K**, **L** NADPH (K) and Rib-5-P concentration (**L**) were measured in *WT* or 6PGL^*−/*−^ A549 and H1299 cells with or without YAP overexpression. The data are shown as the mean ± SD from three biological replicates (including IB). Data in **B**–**E**, **G**–**I**, **K**, **L** were analyzed using a one-way ANOVA test. **P < 0.01, NS nonsignificant.

Existing studies have shown that YAP can activate glycolysis and hexosamine biosynthesis pathways in tumors [14, 15], while whether PPP, another cancer-promoting glucose metabolism pathway, is regulated by YAP has not been clarified. We observed that NADPH and ribose-5-phosphate (Rib-5-P), two key metabolites of PPP, were positively regulated by YAP overexpression (Fig. 4A–C). Furthermore, YAP upregulated the expression of 6PGL, but had no effect on other key metabolic enzymes in PPP pathway (Fig. 4D–F). Overexpression of 6PGL could recover the inhibition of NADPH and Rib-5-P by YAP knockout (Fig. 4G, H, Fig. S4A). The above data suggested that the promotion role of YAP to PPP was exerted through 6PGL. We also revealed that 6PGL overexpression partially reversed YAP knockout-resulted decreased colony formation, cell viability, 3D cell growth, and increased cell apoptosis (Fig. 4I, J, Fig. S4B–D). In addition, 6PGL knockout-induced NADPH and Rib-5-P decline could not be prevented by YAP overexpression (Fig. 4K, L, Fig. S4E). These data suggested YAP acted as the upstream of 6PGL to stimulate PPP.

### YAP boosts the transcription of 6PGL

**Fig. 5:**
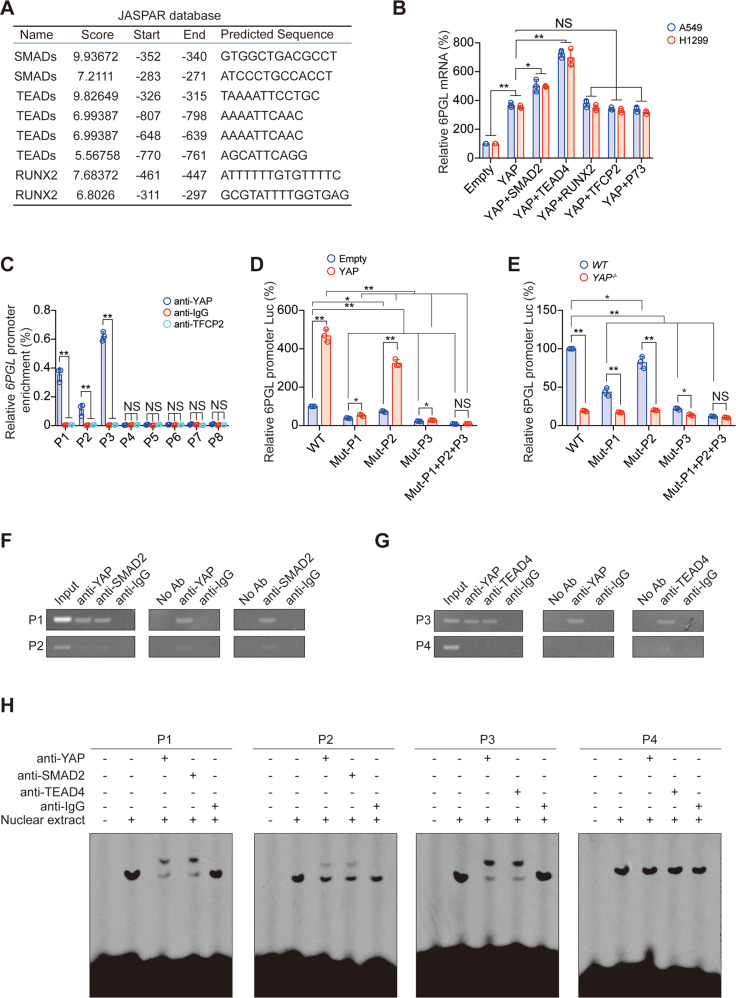
SMAD and TEAD stimulate 6PGL transcription in a YAP-dependent manner. **A** Predicting 6PGL promoter binding YAP-associated transcription factors by JASPAR database. **B** 6PGL mRNA was measured in A549 and H1299 cells with indicated plasmids overexpressed. **C** The enrichments of YAP at indicated regions of the 6PGL promoter were calculated as the percentage of Input chromosomal DNA via ChIP in A549 cells. Anti-IgG and anti-TFCP2 were used as parallel control. **D**, **E** Luciferase activity was analyzed in A549 cells co-expressing indicated 6PGL promoter–reporter with or without overexpressing (**D**) or knocking out YAP (**E**). **F**, **G** Co-occupancies of YAP and SMAD2 (**F**) and YAP and TEAD4 (**G**) were measured by ChIP and Re-ChIP experiments in A549 cells using indicated antibodies. **H** Nuclear extracts from A549 cells were incubated with indicated 6PGL promoter probes with or without the presence of indicated antibodies. The DNA–protein interactions were measured using EMSA. The data are shown as the mean ± SD from three biological replicates (including IB). Data in **B**–**E** were analyzed using a one-way ANOVA test. **P < 0.01, *P < 0.05, NS nonsignificant.

YAP usually acts as a transcription cofactor, promoting the binding of transcription factors to the promoter region of target genes, thereby promoting the transcription of target genes [32]. We used the JASPAR database to predict whether common YAP-associated transcription factors (including SMADs, TEADs, RUNX2, TFCP2, P73) could bind to the promoter region of 6PGL. The data revealed possible binding sites for SMADs, TEADs, and RUNX2 on the 6PGL promoter (Fig. 5A). In addition, SMAD2 and TEAD4 had an additional effect on the elevation of 6PGL mRNA level by YAP, while TFCP2, P73, and RUNX2 had no similar effect (Fig. 5B). Chromatin immunoprecipitation (ChIP) experiments suggested that YAP could bind to two SMADs-related peaks (P1 and P2) and one TEADs-related (P3) peak, but could not bind to the other five peaks (Fig. 5C). The mutation of P1, P2, or P3 significantly inhibited the activity of the 6PGL promoter and the promotion effect of YAP on the 6PGL promoter, and simultaneous mutations of P1–P3 had an additional effect compared to the single mutations of them (Fig. 5D, E). We also performed Re-ChIP and electrophoretic mobility shift assay (EMSA) experiments to reveal that YAP and SMAD2 bound to P1 and P2 simultaneously, while YAP and TEAD4 bound to P3 simultaneously (Fig. 5F–H). In addition, we observed that simultaneous knockout of SMADs and TEADs had additional effects on inhibiting the mRNA and promoter activity of 6PGL compared to the single knockout of one of them, and this inhibitory function could not be reversed by YAP overexpression (Fig. S5A–C). However, when YAP was knocked out, 6PGL mRNA level and promoter activity were significantly reduced and could not be rescued by overexpression of TEAD4 and SMAD2 (Fig. S5D, E). Therefore, these data suggested that the promoter of 6PGL was affected by both SMAD2 and TEAD4 in a YAP dependent-manner.

### YAP ISGylation is essential for its regulation of 6PGL

**Fig. 6:**
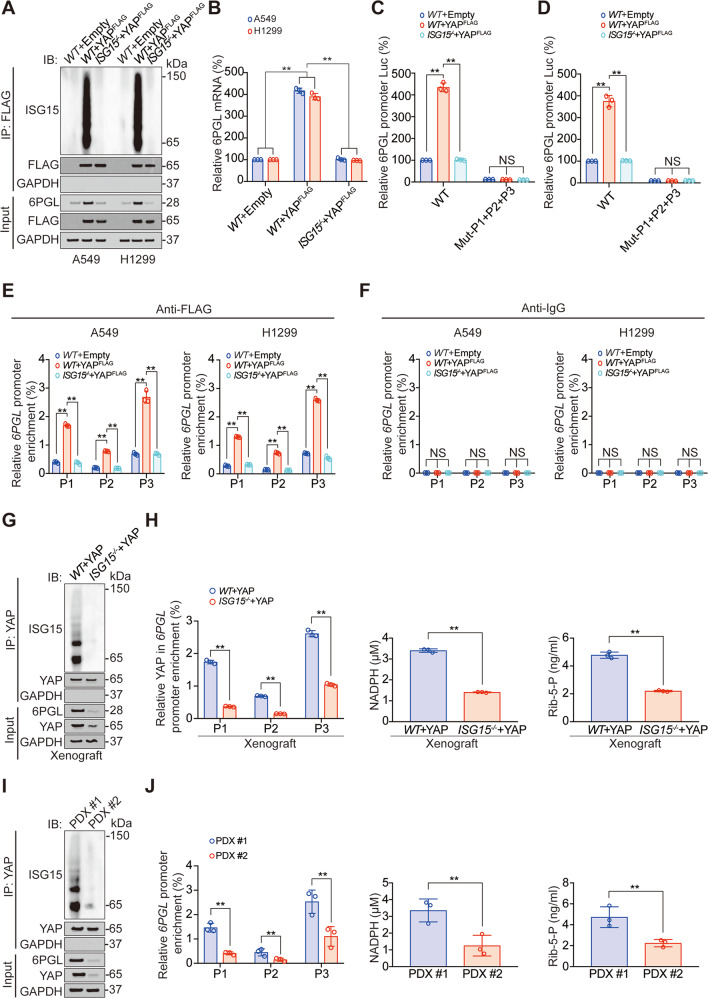
YAP ISGylation stimulates 6PGL transcription. **A** Co-IP experiments analyzing YAP ISGylation in *WT* or *ISG*^−*/*−^ A549 and H1299 cells with or without overexpressing YAP, and YAP and 6PGL expression in Input sample were analyzed by IB. The FLAG level in YAP^FLAG^ overexpressed co-IP samples was adjusted to the same protein content. **B** 6PGL mRNA level was measured in *WT* or *ISG*^*−/*^^−^ A549 and H1299 cells with or without overexpressing YAP. **C**, **D** Luciferase activity was analyzed in *WT* or *ISG15*^*−/−*^ A549 (**C**) and H1299 (**D**) cells co-expressing indicated 6PGL promoter–reporter with or without overexpressing YAP. **E**, **F** The enrichments of YAP at indicated regions of 6PGL promoter were calculated as the percentage of Input chromosomal DNA via ChIP in *WT* or *ISG*^*−/−*^ A549 and H1299 cells with or without overexpressing YAP (**E**). Anti-IgG was used as parallel control (**F**). **G** Co-IP experiments analyzing YAP ISGylation in tumor xenografts overexpressing YAP with or without ISG15 knockout, and YAP and 6PGL expression in Input sample were analyzed by IB. The YAP level in each co-IP sample was adjusted to the same protein content. **H** The enrichments of YAP at indicated regions of 6PGL promoter, NADPH, and Rib-5-P level were analyzed in the same tumor xenografts as those in Panel G. **I** Co-IP experiments analyzing YAP ISGylation in PDX#1 and PDX#2, and YAP and 6PGL expression in Input sample were analyzed by IB. The YAP level in each co-IP sample was adjusted to the same protein content. **J** The enrichments of YAP at indicated regions of 6PGL promoter, NADPH, and Rib-5-P level were analyzed in PDX#1 and PDX#2. The data are shown as the mean ± SD from three biological replicates (including IB). Data in **B**–**F** were analyzed using a one-way ANOVA test. Data in **H** and **J** were analyzed using a student’s *t* test. **P < 0.01, NS nonsignificant.

Since YAP ISGylation is essential for its pro-transcription function (Fig. 2), we subsequently investigated whether the stimulating role of YAP on 6PGL transcription depends on its ISGylation. We observed that knockout of ISG15 reversed YAP-induced 6PGL elevation in protein and mRNA level (Fig. 6A, B), as well as promoter activity (Fig. 6C, D). However, simultaneous mutations of P1–P3 abolished 6PGL promoter activity, and 6PGL promoter activity was no longer regulated by ISG15 (Fig. 6C, D). Moreover, YAP bindings to P1–P3 of the 6PGL promoter were also held back by ISG15 knockout (Fig. 6E, F). We used the previously described CDX and PDX models in Fig. 3A–I, and found in CDX models, ISG knockout significantly inhibited 6PGL expression, YAP bindings to P1–P3 of 6PGL promoter, NADPH, and Rib-5-P level (Fig. 6G, H). Similarly, in PDX#1 model (high YAP ISGylated model), 6PGL expression, YAP bindings to P1–P3 of 6PGL promoter, NADPH and Rib-5-P level were significantly higher than PDX#2 model (low YAP ISGylated model) (Fig. 6I, J). We also found in previously reported 20 primary cell lines in Fig. 3J–L, YAP ISGylation level was positively associated with 6PGL expression, NADPH, and Rib-5-P level (Fig. S6A–C). Collectively, these data indicated YAP ISGylation was critical for YAP-induced 6PGL transcription.

### Clinical association among YAP ISGylation, YAP, and 6PGL level

**Fig. 7:**
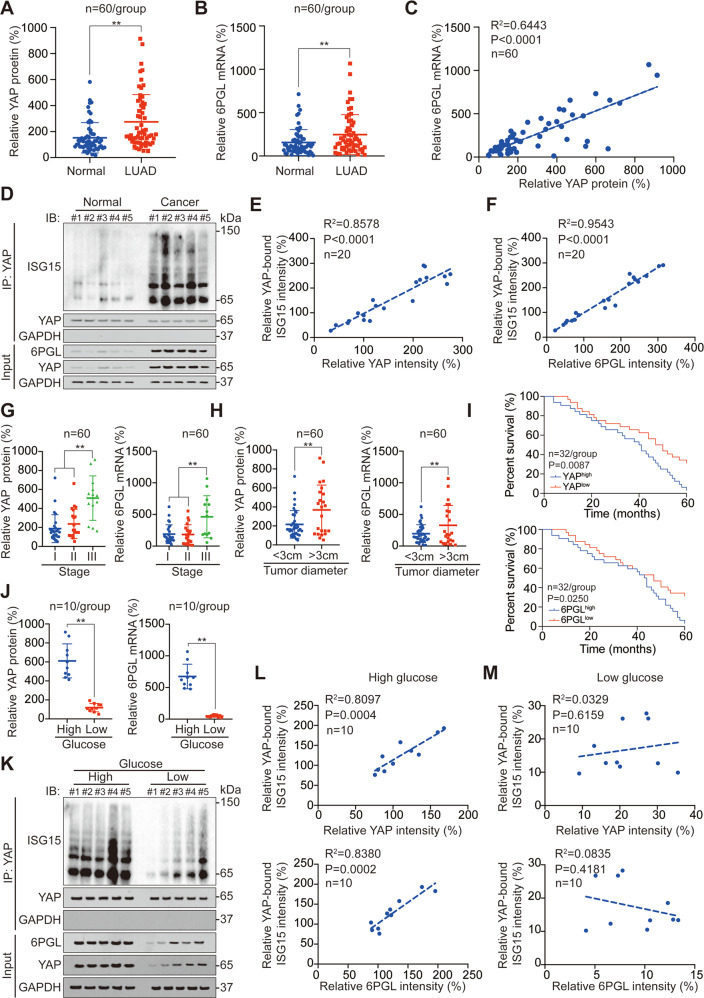
Clinical association among YAP ISGylation, YAP, and 6PGL. **A** YAP protein level in LUAD and adjacent normal tissues as measured by ELISA. **B** 6PGL mRNA level in LUAD and adjacent normal tissues as measured by qPCR. **C** Association between YAP protein and 6PGL mRNA level. **D** Co-IP experiments analyzing YAP ISGylation in LUAD and adjacent normal tissues, and YAP and 6PGL expression in Input sample were analyzed by IB. The YAP level in each co-IP sample was adjusted to the same protein content. **E**, **F** Association between YAP bound ISG15 intensity and YAP intensity (**E**), as well as YAP, bound ISG15 intensity and 6PGL intensity (**F**) in LUAD tissues. **G**, **H** YAP protein and 6PGL mRNA level in stage I–III LUAD samples (**G**), <3 cm and >3 cm diameter LUAD samples (**H**). **I** Survival analysis for LUAD patients with high or low YAP protein and 6PGL mRNA level. **J** YAP protein and 6PGL mRNA level in high and low glucose LUAD samples. **K** Co-IP experiments analyzing YAP ISGylation in high and low glucose LUAD tissues, and YAP and 6PGL expression in Input sample were analyzed by IB. The YAP level in each co-IP samples was adjusted to the same protein content. **L**, **M** Association between YAP bound ISG15 intensity and YAP intensity, as well as YAP, bound ISG15 intensity and 6PGL intensity in high (**L**) or low (**M**) glucose LUAD tissues. The data are shown as the mean ± SD from three biological replicates (including IB). Data in **A**, **B**, **H**, **I** were analyzed using a student’s *t* test. Data in **C**, **E**, **F**, **L**, **M** were analyzed using the Spearman rank correlation analysis. Data in **G** were analyzed using a one-way ANOVA test. Data in **I** were analyzed using a log-rank test. **P < 0.01.

Subsequently, we analyzed the clinical associations of the factors in this study. After analyzing 60 paired tissues, we found that YAP protein (by ELISA) and 6PGL mRNA levels were significantly higher in LUAD tissues compared to adjacent normal tissues, and a positive correlation between 6PGL mRNA and YAP protein in LUAD tissues was also revealed (Fig. 7A–C). We also analyzed YAP and 6PGL protein as well as YAP ISGylation level in tissues using IB and found that they were all elevated in LUAD tissues compared to their adjacent normal tissues (Fig. 7D, Fig. S7A–C). Positive associations between YAP ISGylation and YAP protein level, as well as 6PGL protein level, were also revealed (Fig. 7E, F). Moreover, YAP protein and 6PGL mRNA levels were upregulated in stage III LUAD tissues compared to stage I and II LUAD tissues (Fig. 7G), as well as in >3 cm diameter LUAD tissues compared to <3 cm LUAD tissues (Fig. 7H). We also found that high expression of YAP protein and 6PGL mRNA were associated with poor LUAD prognosis (Fig. 7I). As a histological marker, YAP protein has an area under the receiver operating characteristic curve (AUC-ROC) of 0.714, a sensitivity of 71.7%, and a specificity of 65.0%. The AUC-ROC of 6PGL mRNA as a histological marker was 0.629, sensitivity was 62.3%, and specificity was 61.7% (Fig. S7D). In addition, neither YAP and 6PGL protein or mRNA could be detected in serum (Fig. S7E).

Since YAP is a positive regulator for glycolysis, PPP, and HBP in stimulating glucose metabolism (Figs. 4–6 [14, 15]), we further analyzed YAP and 6PGL level as well as YAP ISGylation in different blood glucose level tissues. We observed that YAP protein, 6PGL mRNA, and YAP ISGylation were all significantly elevated in high glucose LUAD tissues compared to low glucose LUAD tissues (Fig. 7J, K, Fig. S7F). In addition, YAP ISGylation level was significantly associated with YAP and 6PGL levels in high glucose LUAD tissues (Fig. 7L), whereas in low glucose LUAD tissues, no significant correlations were uncovered among YAP ISGylation, YAP, and 6PGL level (Fig. 7M). We also found that although blood glucose was significantly elevated in stage III LUAD patients compared to stage I and II patients (Fig. S7G), no significant correlation was found between blood glucose and LUAD stage (Fig. S7H). These data suggested that YAP ISGylation, YAP, and 6PGL level were positively associated with each other, especially in high glucose LUAD tissues.

## Discussion

YAP is regulated by a variety of post-translational modifications. It is best known that YAP is phosphorylated at Ser127 (Ser, serine) and then inhibited [20]. Our Lab first reported the YAP O-GlcNAcylation, which inhibits YAP phosphorylation, promotes YAP to enter the nucleus, and activates downstream transcription [15]. Here, we observed that YAP could undergo a kind of ubiquitination-like modification, ISGylation. In this study, we identified the possible YAP ISGylation site as K497. This ISGylation inhibited ubiquitination of YAP at the same site, further inhibited YAP proteasome degradation, and promoted downstream transcription. These findings further enrich the theory that YAP is regulated by post-translational modification.

Initially, it is thought that YAP is mainly regulated by the Hippo pathway [33]. However, recently, some regulations of YAP are found to be independent of upstream Hippo signaling. For example, enhanced YAP O-GlcNAcylation stimulates YAP to enter the nucleus and promote downstream transcription [15, 34]. Moreover, CREB is identified by our Lab as a transcription factor up-regulating YAP mRNA level [35]. In addition, erb-b2 receptor tyrosine kinase 2 (ERBB2) is also identified as an upstream transcription factor of YAP [36]. The YAP ISGylation was also independent of the Hippo pathway and enhanced the stability of YAP by directly inhibiting its ubiquitination. All the above studies indicate that in addition to the Hippo pathway, YAP is also regulated by many other ways, which are also crucial to the regulation of YAP [3, 37].

YAP is a crucial regulator of glucose metabolism. As for glycolysis, YAP can directly promote the transcription of phosphoglycerate mutase 1 (PGAM1) or promote the transcription of pyruvate kinase-2 (PKM-2) through hypoxia-inducible factor 1 subunit alpha (HIF-1α) to activate glycolysis [14, 38]. Moreover, YAP stimulates HBP by activating the transcription of two key enzymes, nudix hydrolase 9 (NUDT9) and solute carrier family 5 member 3 (SLC5A3) [15]. In this study, we observed that YAP was also a stimulator for PPP. Without regulating other enzymes, YAP stimulated the transcription of 6PGL through SMAD2 and TEAD4. The influence of transcription factors on the target gene promoter is very complex, and one promoter can be affected by multiple transcription factors at the same time [39, 40]. In our study, it was found that the transcription of 6PGL could not be activated by overexpression of TEAD4 and SMAD2 after knocking out YAP. Therefore, we speculated that if we want to inhibit the transcription of an oncogene in clinical treatment, it is not feasible to inhibit the direct transcription factors, because the oncogene will be activated by multiple transcription factors at the same time, while suppressing transcription cofactors like YAP can achieve the purpose of simultaneously inhibiting multiple transcription factors.

YAP and high glucose have mutually promoting feedback effects, YAP can generate a large number of intermediate products needed for tumor proliferation through glycolysis, PPP, and HBP, while high glucose can activate YAP such as promoting its O-GlcNAcylation and interaction with TEADs [14, 15, 38, 41]. In this study, we found that YAP protein and 6PGL mRNA had certain diagnostic effects as tissue markers, but their optimal diagnostic specificities and sensitivities were all less than 75%, and they could not be detected in blood without trauma, so they were of little significance for early diagnosis (Figure S7D-E). Factors downstream of YAP, such as membrane protein melanoma cell adhesion molecule, chaperonin containing t-complex 1 subunit 3, circRNA104075 which are blood detectable indicators, can indeed play a certain role in the early diagnosis of tumors [42–44]. However, due to the high expression of YAP in a variety of tumors, the diagnostic specificity of YAP-related indicators is somewhat deficient [1, 45, 46], and their sensitivity in the diagnosis of LUAD is not as good as that of low-dose spiral CT at present [47]. Therefore, we believed that it may be more meaningful to further explore the role of YAP in prognostic monitoring and as an individual therapeutic target, or to integrate YAP-related markers into a multi-marker panel. In this study, we further demonstrated that YAP is highly expressed and ISGylated in patients with hyperglycemic cancer. Therefore, targeting YAP and its ISGylation for hyperglycemic tumors may achieve promising therapeutic effects. This is not only limited to LUAD discussed in this study, but may also include liver, colorectal, breast, pancreatic cancer, and other tumors that are more closely related to hyperglycemia [48]. Therefore, further research on YAP promoting tumors through glucose metabolism is needed, which is conducive to the proposal of individualized tumor diagnosis and treatment.

## Conclusions

YAP ISGylation is critical for maintaining its stability and further activation of PPP. Targeting ISGylated YAP might be promising for hyperglycemia cancer treatment.

## Material and methods

### Cell culture

Established A549 and H1299 cell lines were purchased from Fuheng Biotechnology (Shanghai, China). All the cell lines were validated by short tandem repeat analysis. Patient-derived primary LUAD cells were established from LUAD tissues as previously described [49]. Briefly, fresh tissues less than 1.0 cm^3^ without necrosis were immediately rinsed 3 times with cold Dulbecco’s Phosphate Buffered saline and then re-suspended in Dulbecco’s Modified Eagle Medium (DMEM) containing collagenase I (2 mg/ mL, Solarbio, Shanghai) at 37 °C for 4 h. After being rinsed with DMEM 3 times, cells were cultured at 37 °C, 5% CO_2_ condition. For monolayer culture, cells were cultured in DMEM containing 10% fetal bovine serum and 1% penicillin/streptomycin. For 3D spheroid culture, basement membrane extract (BME) (Trevigen, Gaithersburg, MD, USA) was seeded in a 96-well plate at 50 μl/well and pre-warmed at 37 °C for 0.5 h. Then, cells were seeded on top of the plate coated with BME at a density of 10^5^ cells per well. Images were captured using a microscope [49].

### Mouse experiments and tissue samples

For a generation of CDX mouse models, A549 cells (initial 5 × 10^6^, including control, YAP overexpression, ISG15 knockout with YAP overexpression cells) were subcutaneously injected into 6-week-old athymic nude mice (Jiesijie, Shanghai, China). For a generation of PDX mouse models, fresh LUAD specimens (high YAP and ISGylation level as PDX#1, low YAP, and ISGylation level as PDX#2) in a size of 2–3 mm^3^ were implanted into six-week-old athymic nude mice. The third generation of PDX-bearing mice was used for further analysis [49]. The tumor volume was calculated as 0.5 × *L* × *W*^2^ (*L* indicating length while *W* indicating width). All the tissue and serum specimens were recruited in Shanghai Chest Hospital (Shanghai, China) (mean age ± SD, 63.91 ± 11.27 years; male: female ratio, 1.17:1) were recruited in Shanghai Chest Hospital (Shanghai, China) from March 2015 to December 2020. Informed written consents were obtained from all patients.

### Regents and plasmids

For regents, CHX (Sigma, St Louis, MO, USA), MG132 (MedChemExpress, Monmouth Junction, NJ, USA), 3-MA (MedChemExpress), CHQ (MedChemExpress), Bort (MedChemExpress), and IFNα (Sigma) were used to treat cells. For plasmids, ISG15 and 6PGL expression plasmids were bought from Origene (Beijing, China). LentiCRISPR v2 based constructs were used for knockout ISG15, UbCH8, HERC5, 6PGL, SMADs, TEADs. pGL4.21 vector was used to construct a 6PGL promoter-luciferase vector. YAP^K280R-HA^, YAP^K321R-HA^, YAP^K497R-HA^, 6PGL^Mut-P1^, 6PGL^Mut-P2^, 6PGL^Mut-P3^, and 6PGL^Mut-P1+P2+P3^ mutant plasmids were constructed using overlapping PCR. YAP, ATG5, PSMB5, and βTrCP knockout constructs, YAP^WT-HA^, YAP^FLAG^, SMAD2, TEAD4, RUNX2, TFCP2, P73, pUAS-Luc/TEAD-Gal4 plasmids were acquired from previous studies [15, 31, 43, 49, 50]. The primers are listed in Supplementary Table 1.

### Immunofluorescence (IF), immunohistochemistry (IHC), immunoblotting (IB), and enzyme-linked immunosorbent assay (ELISA)

IF and IHC were performed according to the conventional protocols. The primary antibodies used for IF were anti-YAP (Abcam, Hong Kong, China #ab52771) and anti-βTrCP (Abcam, #ab233638). The primary antibodies used for IHC were: anti-YAP (Santa Cruz Biotechnology, Santa Cruz, CA, USA, #sc-101199) and anti-ISG15 (Abcam, #ab233071). For IB, the proteins were resolved on SDS-PAGE gels according to the conventional protocols. The primary antibodies used were anti-ISG15 (Abcam, #ab233071), anti-YAP (Abcam, #ab52771 and Santa Cruz, #sc-101199), anti-GAPDH (CST, #5174 and #51332), anti-Ub (Abcam, #ab7780 and #ab7254), anti-PSMB5 (Abcam, #ab167341), anti-βTrCP (Abcam, #ab71753 and #ab233638), anti-YAP^O241^ (developed by Biolynx, Hangzhou, China), anti-YAP^P127^ (Abcam, #ab76252), anti-YAP^P397^ (CST, Boston, MA, USA, #13619), anti-HA (Abcam, #ab9110 and #ab18181), anti-TEAD4 (Abcam, #ab197589 and #ab58310), anti-6PGL (Abcam, #ab229872), anti-FLAG (CST, #8146 and #2368), anti-UbCH8 (Abcam, #ab177485), anti-HERC5 (Invitrogen, Carbsland, CA, USA, #703675), anti-ATG5 (Abcam, #ab221604), anti-LATS1 (Abcam, #ab243656), anti-CK1 (Abcam, #ab270997 and #ab115293), anti-SMAD2 (Abcam, #ab40855), anti-alpha fetoprotein (AFP, Abcam, #ab284388) and anti-albumin (Alb, Abcam, #ab207327). ELISA kits (Yingxin, Shanghai, China) were used to measure the concentration of YAP protein and Rib-5-P.

### Co-immunoprecipitation (co-IP)

Co-immunoprecipitation (co-IP) was performed as described previously [31]. After cells were harvested, the lysates were mixed with 50 ul of protein A/G-magnetic beads (Novex, Oslo, Norway) and incubated at 4 °C overnight with the indicated antibodies cross-linked to protein A/G magnetic beads. The beads were washed three times with Western/IP lysis buffer (Beyotime, Haimen, China), suspended in SDS-PAGE loading buffer, and then detected by IB with relevant antibodies. The antibodies used for co-IP were: anti-YAP (Abcam, #ab52771 and Santa Cruz, #sc-101199), anti-βTrCP (Abcam, #ab71753), anti-HA (Abcam #ab18181), anti-FLAG (CST, #2368), anti-CK1 (Abcam, #ab270997), anti-TEAD4 (Abcam, #ab197589) and anti-LATS1 (Abcam, #ab234820).

### Proteasome isolation

Proteasomes were isolated using the proteasome isolation kit (Sigma, #539176). Isolation was performed strictly in accordance with guidelines provided by the manufacturer. Affinity and control beads were used to isolate the proteasome and serve as a negative control.

### Proximity ligation assay

PLA was performed using the Duolink In Situ Red Starter Kit (Sigma) as previously described [31]. Cells were seeded on glass coverslips in 24-well plates. On the second day, the cells were fixed with 4% PFA and blocked with a blocking buffer. Then, the cells were incubated overnight at 4 °C in suitable primary antibodies. The primary antibodies used were anti-YAP (Abcam, China #ab52771) and anti-βTrCP (Abcam, #ab233638).

### Measurements of metabolites, luciferase activity, cell viability, caspase 3/7 activity, and anchorage-independent colony formation

NADPH (Sigma) concentration was measured using the appropriate kits according to the manufacturer’s instructions. Luciferase activities were measured using a dual-luciferase reagent (Promega, Madison, WI, USA). Cell viability was measured using a CCK8 kit (Beyotime). Caspase3/7 activity was determined using Caspase 3/7 Glo luciferase reagent (Promega). As for the anchorage-independent colony formation assay, LUAD cells were seeded in a 6-well plate containing 0.3% agarose in DMEM at a density of 6 × 10^3^ cells per well. Two weeks later, the numbers of colonies were calculated under a microscope.

### Quantitative RT-PCR (qPCR)

Total RNA was extracted using Trizol (Ambion, Carlsbad, CA, USA) and reverse-transcribed into complementary DNA using the PrimeScript^TM^ RT reagent kit (Takara, Dalian, China). The SYBR premix Ex Taq (Takara) kit was used for real-time qPCR. For the evaluation of the mRNA level of YAP and 6PGL in serum, semi-qPCR was performed. The PCR was terminated at cycle 29 and the products were visualized by agarose gel electrophoresis. The primers are listed in Supplementary Table 1.

### ChIP and Re-ChIP

ChIP and Re-ChIP experiments were performed using the kits from Active Motif (Carlsbad, CA, USA) as previously described [31], and strictly in accordance with guidelines provided by the manufacturer. The primary antibodies used in ChIP and Re-ChIP experiments were: anti-YAP (CST, #14074), anti-TFCP2 (CST, #80784), anti-SMAD2 (Abcam, #ab40855), anti-TEAD4 (Abcam, #ab155244) and anti-IgG (CST, #3900). The primers are listed in Supplementary Table 1.

### EMSA

EMSA was performed as described in the previous study [49]. The light shift kit (Pierce, Rockford, IL, USA) was used. Nuclear extracted proteins were prepared using the kit from Active Motif (Carlsbad) and incubated in the reaction buffer on ice followed by the addition of the biotin-labeled probes (synthesized and 5′ labeled by Sangon Inc., Shanghai, China). For supershift assays, antibodies against YAP (CST, #14074), TEAD4 (Abcam, #ab155244), and SMAD2 (Abcam, #ab40855) were added to the mixture before adding the probe. The probes used are listed in Supplementary Table 1.

### Statistical analysis

Tests used in this study included student’s *t* test, one-way, two-way ANOVA log-rank, *χ*^2^ test, and the Spearman rank correlation analysis. A *P* < 0.05 was considered statistically significant.

## Supplementary information

Supplementary Figures

Supplementary Table

## Data Availability

The data that support the findings of this study are available from the corresponding author upon reasonable request.
