# Supplementary material for: YAP ISGylation increases its stability and promotes its positive regulation on PPP by stimulating 6PGL transcription
Source: Cell Death Discov. 2022 Feb 11;8:59. doi: 10.1038/s41420-022-00842-8 (PMC8837792; doi:10.1038/s41420-022-00842-8)
Supplement: Supplementary file 1 — Supplementary Figures [file 41420_2022_842_MOESM1_ESM.docx]

**Supplementary Figures**

**Supplementary Figure 1**


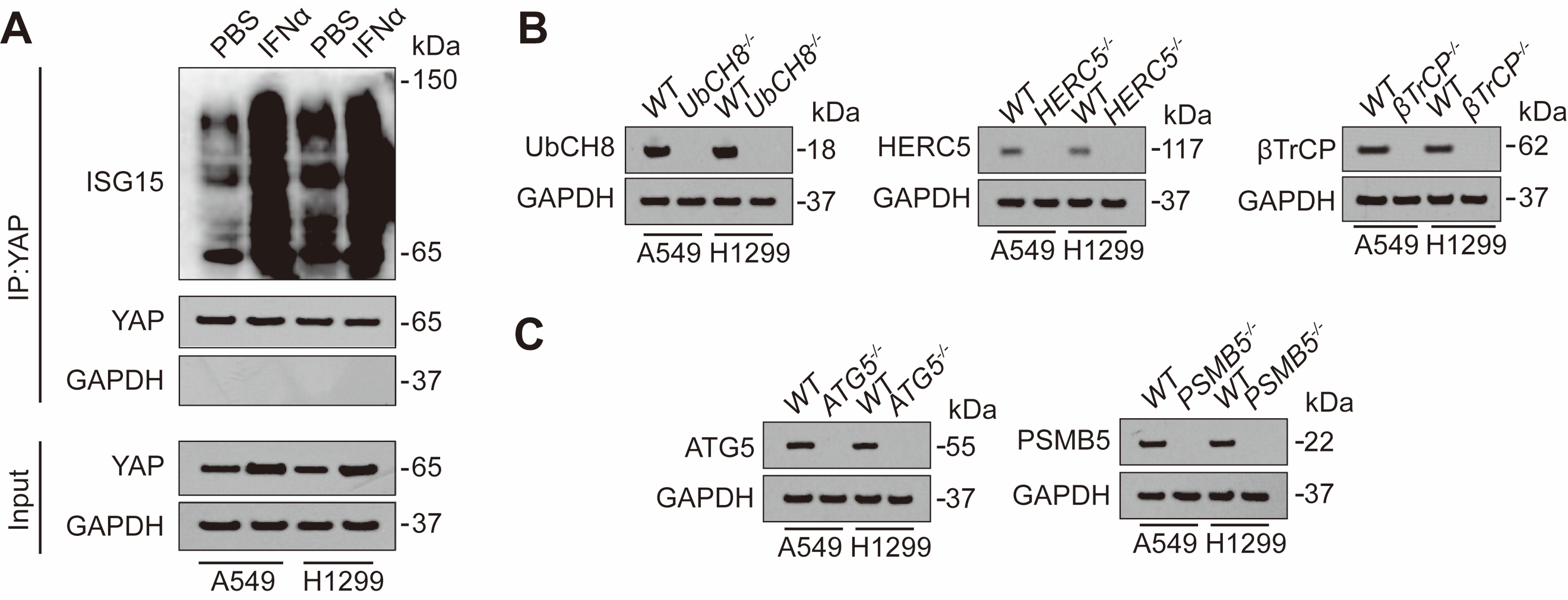


**Supplementary Figure 1. Supplementary to Figure 1.**

(A) Co-IP was performed in PBS or IFNα (1000 IU/ml) treated A549 and H1299 cells using anti-YAP antibodies. The YAP level in each co-IP samples was adjusted to the same protein content. Indicated proteins were further analyzed by IB.

(B-C) UbCH8, HERC5, βTRCP (B), ATG5 and PSMB5 (C) expression were detected by IB in UbCH8, HERC5, βTRCP (B), ATG5 and PSMB5 (C) knockout cells. The data are shown from three biological replicates.

**Supplementary Figure 2**


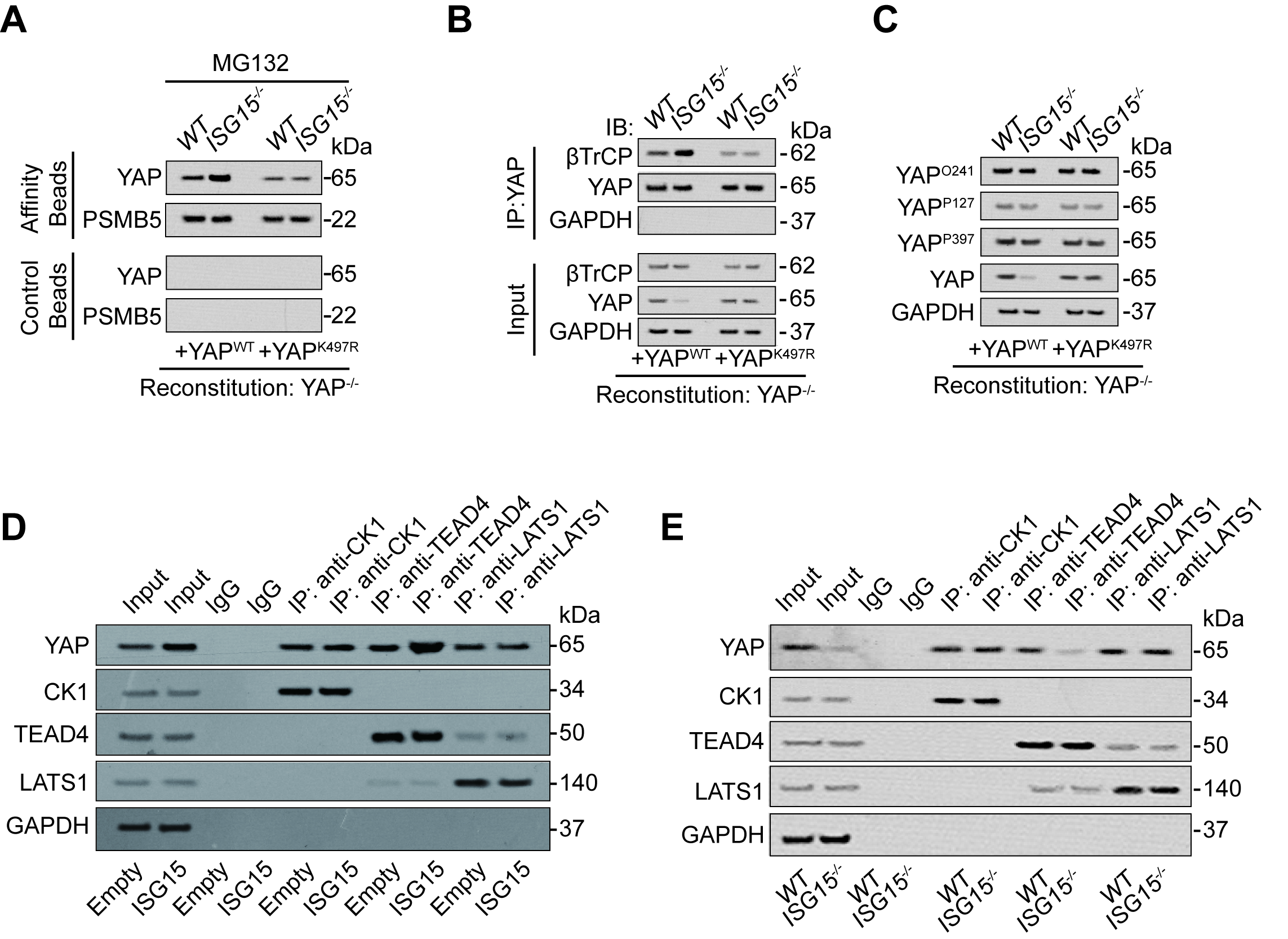


**Supplementary Figure 2. Supplementary to Figure 2.**

(A) Association of YAP and PSMB5 analyzed by IB in proteasomes isolated from YAP^-/-^ reconstituted A549 cells with or without YAP^WT-HA^ or YAP^K497R-HA^ expressed or ISG15 knocked out in the presence of MG132 (8 μM, 24 h). Samples from affinity or control beads were analyzed in parallel.

(B) Co-IP was performed using anti-YAP antibodies in YAP^-/-^ reconstituted A549 cells with or without YAP^WT-HA^ or YAP^K497R-HA^ expressed or ISG15 knocked out. Indicated proteins were further analyzed by IB. The YAP level in each co-IP samples was adjusted to the same protein content.

(C) O-GlcNAcylation of YAP at T241 (YAP^O241^), phosphorylation of YAP at S127 (YAP^P127^) and S397 (YAP^P397^) were analyzed in YAP^-/-^ reconstituted A549 cells with or without YAP^WT-HA^ or YAP^K497R-HA^ expressed or ISG15 knocked out.

(D-E) Co-IP was performed using indicated antibodies in A549 cells with or without ISG15 overexpression (D) or knockout (E). The target proteins bound by the antibody were adjusted to the same content in each co-IP sample. Indicated proteins were further analyzed by IB.

The data are shown from three biological replicates.

**Supplementary Figure 3**


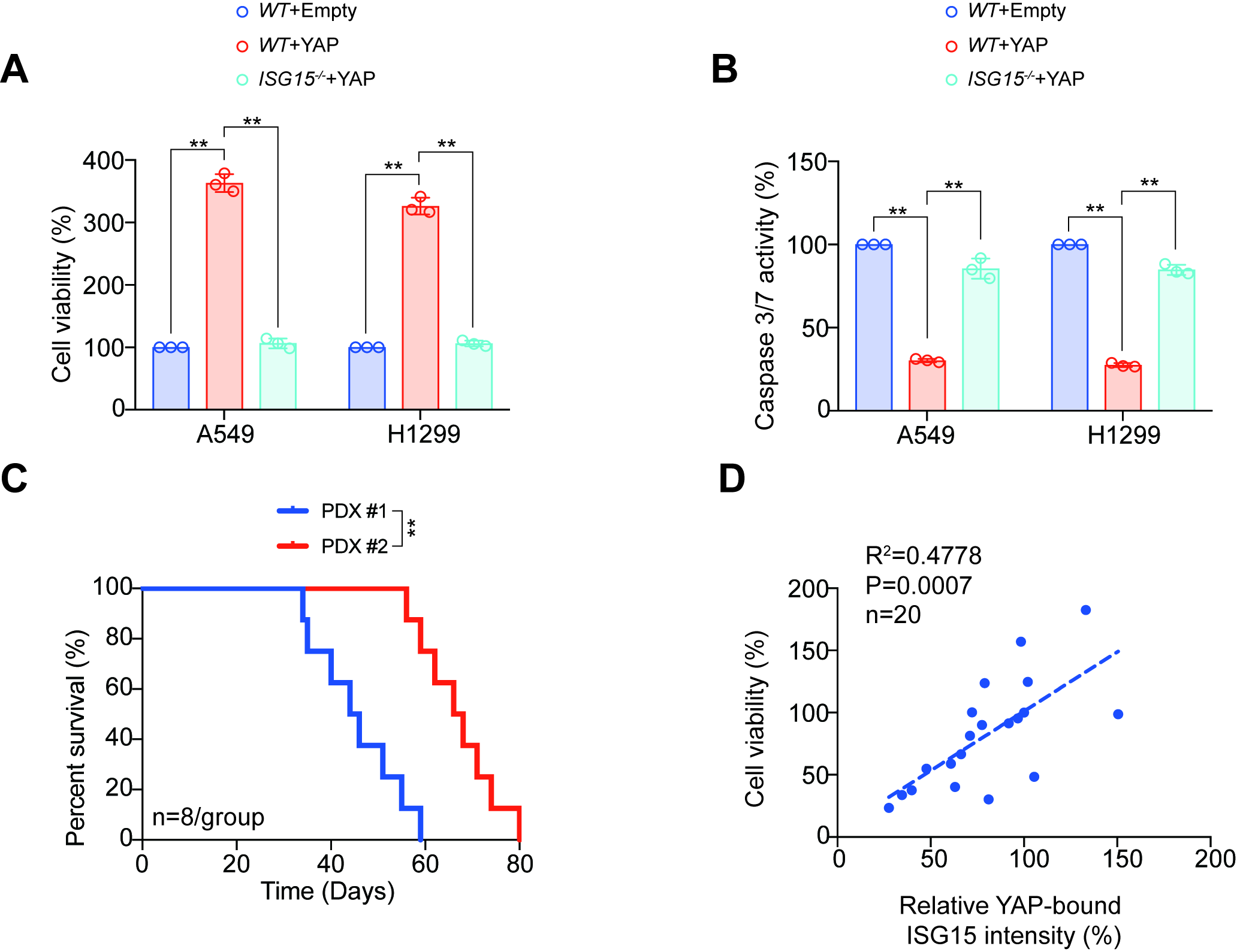


**Supplementary Figure 3. Supplementary to Figure 3.**

(A-B) Cell viability and caspase 3/7 activity were analyzed in *WT or ISG15^-/-^* A549 cells with or without YAP overexpression.

(C) Survival analysis for PDX#1 and PDX#2.

(D) Association between cell viability and YAP-bound ISG15 intensity.

The data are shown as the mean ± SD from three biological replicates. Data in A and B were analyzed using a one-way ANOVA test. Data in C were analyzed using a log rank test. Data in D were analyzed using a Spearman rank-correlation analysis. **, P<0.01.

**Supplementary Figure 4**


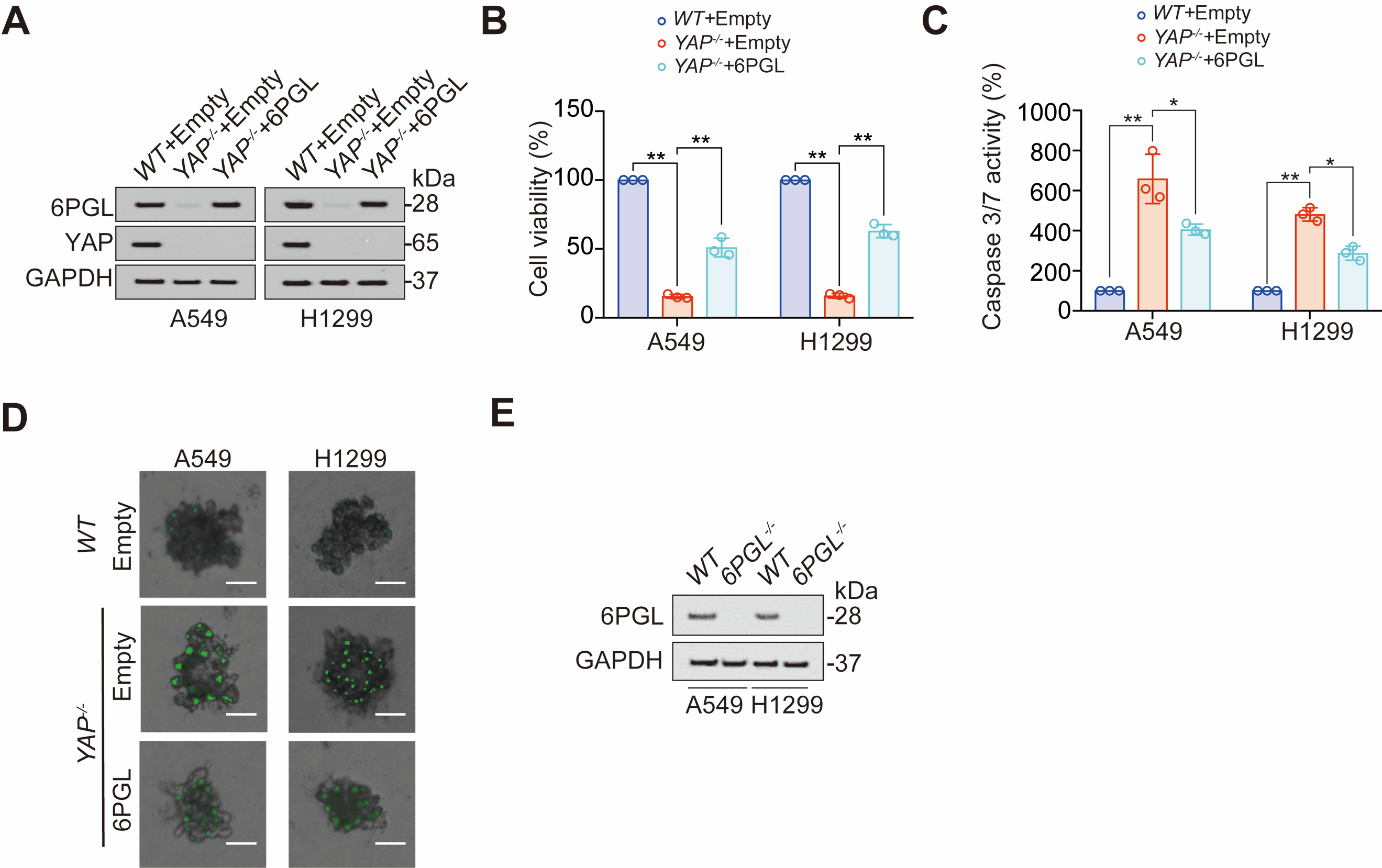


**Supplementary Figure 4. Supplementary to Figure 4.**

(A-D) 6PGL and YAP expression (A), cell viability (B), caspase 3/7 activity (C) and 3D cell growth (D) were measured in *WT* or *YAP^-/-^* A549 and H1299 cells with or without 6PGL overexpression. Scale bar, 50 μm.

(E) 6PGL and YAP expression were measured by IB in *WT* or *6PGL^-/-^* A549 and H1299 cells.

The data are shown as the mean ± SD from three biological replicates (including IB). Data in B-C were analyzed using a one-way ANOVA test. **, P<0.01, *, P<0.05.

**Supplementary Figure 5**


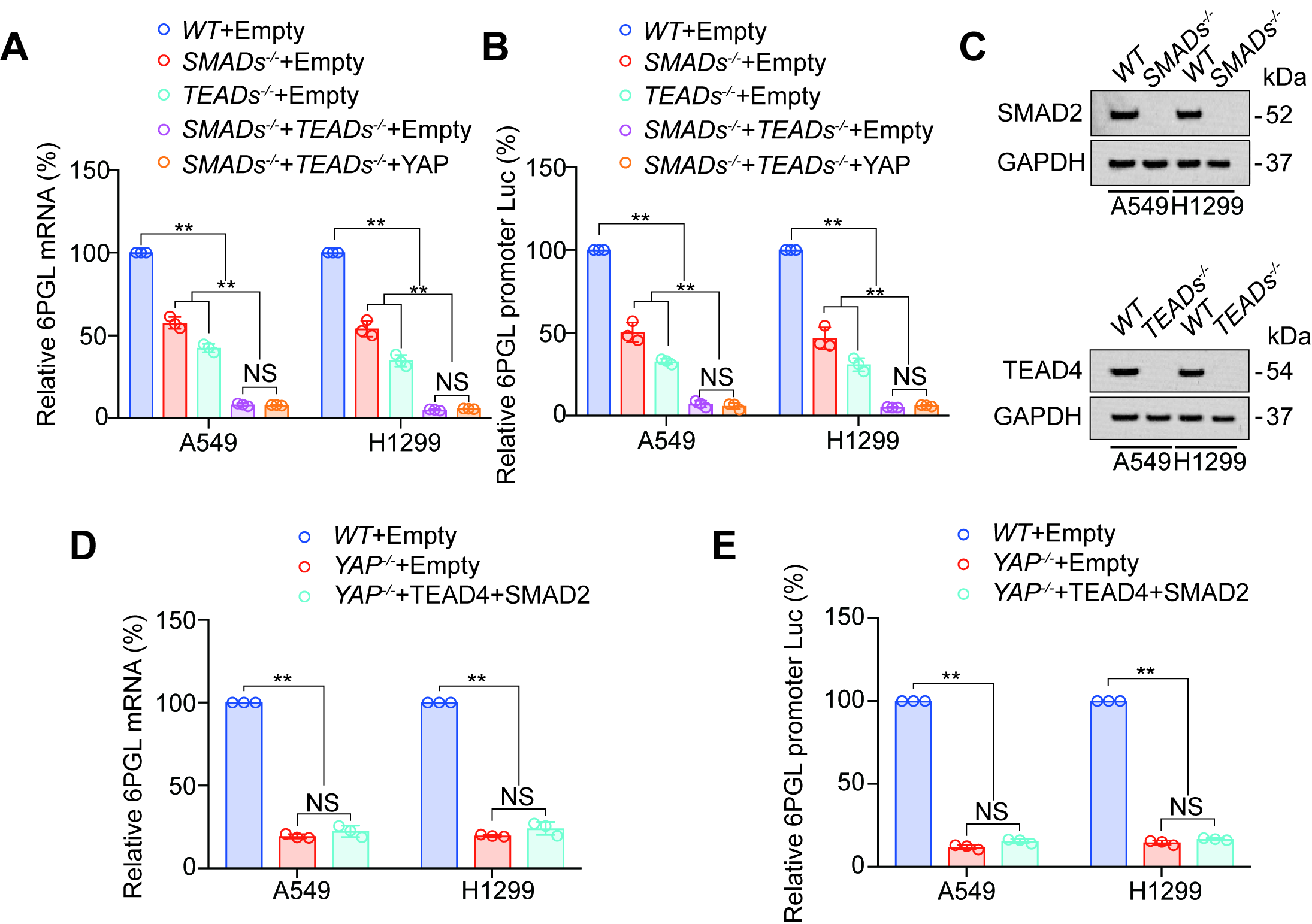


**Supplementary Figure 5. Supplementary to Figure 5.**

(A-B) 6PGL mRNA level (A) and promoter luciferase activity (B) were measured in SMADs or TEADs knockout A549 and H1299 cells with or without YAP overexpression.

(C) SMAD2 and TEAD4 were measured in A549 and H1299 cells with or without SMADs or TEADs knockout.

(D-E) 6PGL mRNA level (D) and promoter luciferase activity (E) were measured in *WT* or *YAP^-/-^* A549 or H1299 cells with or without TEAD4 and SMAD2 overexpression.

The data are shown as the mean ± SD from three biological replicates (including IB). Data in A-B and D-E were analyzed using a one-way ANOVA test. **, P<0.01, NS, nonsignificant.

**Supplementary Figure 6**


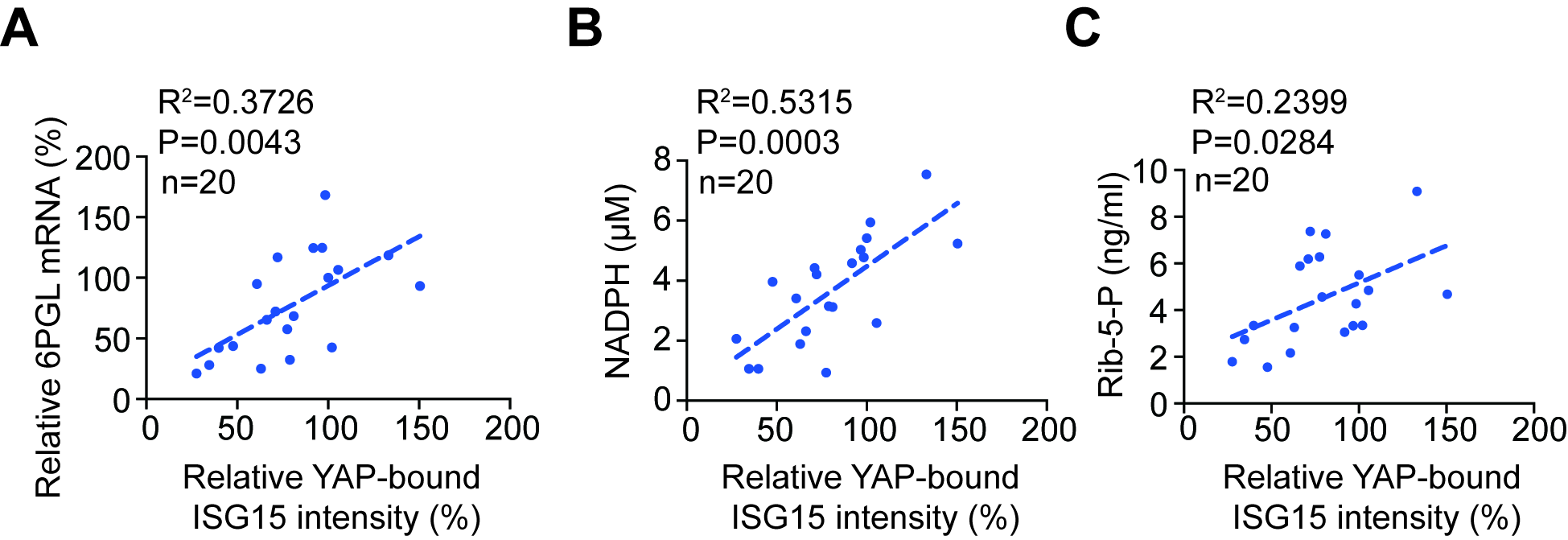


**Supplementary Figure 6. Supplementary to Figure 6.**

(A-C) Association between YAP-bound ISG15 intensity and 6PGL mRNA level (A), NADPH (B) and Rib-5-P concentration (C). Data were analyzed using a Spearman rank-correlation analysis.

**Supplementary Figure 7**


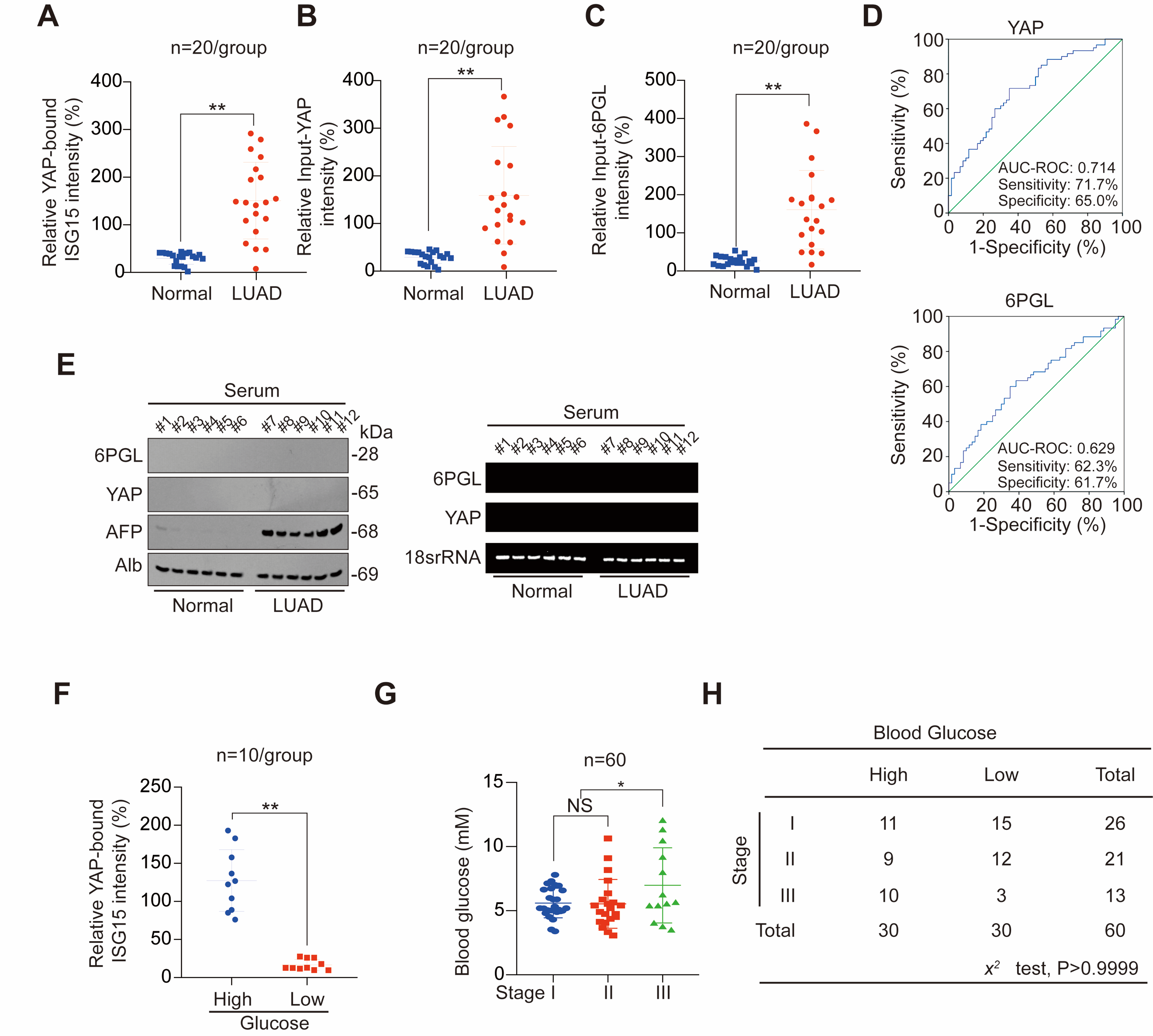


**Supplementary Figure 7. Supplementary to Figure 7.**

(A-C) Relative YAP-bound ISG15 (A), Input-YAP (B) and Input-6PGL intensity (C) in LUAD and adjacent normal tissues.

(D) ROC curves for YAP protein and 6PGL mRNA for the discrimination of LUAD and adjacent normal tissues. The AUC-ROC, sensitivity and specificity were listed.

(E) 6PGL, YAP, AFP and Alb protein were measured by IB, and 6PGL, YAP mRNA and 18srRNA were measured by semi-qPCR in normal and LUAD serum.

(F) Relative YAP-bound ISG15 intensity in high and low glucose LUAD tissues.

(G) Blood glucose level in stage I, II and III LUAD patients.

(H) The correlation between blood glucose level and LUAD stage.

Data in A-C and F were analyzed using a student’s t test. Data in G were analyzed using a one-way ANOVA test. Data in H were analyzed using a χ^2^ test. *, P<0.05, **, P<0.01, NS, nonsignificant.
