# Supplementary material for: YAP ISGylation increases its stability and promotes its positive regulation on PPP by stimulating 6PGL transcription
Source: Cell Death Discov. 2022 Feb 11;8:59. doi: 10.1038/s41420-022-00842-8 (PMC8837792; doi:10.1038/s41420-022-00842-8)
Supplement: Supplementary file 2 — Supplementary Table [file 41420_2022_842_MOESM2_ESM.docx]

**Supplementary Table 1. Primers, sgRNAs and probes in the study.**

| Primers used for qPCR |  |
| --- | --- |
| Name | 5'-3' |
| YAP-qPCR-F | CCTCGTTTTGCCATGAACCAG |
| YAP-qPCR-R | GTTCTTGCTGTTTCAGCCGCAG |
| GAPDH-qPCR-F | ATCATCCCTGCCTCTACTGG |
| GAPDH-qPCR-R | GTCAGGTCCACCACTGACAC |
| CTGF-qPCR-F | CCTGTGCAGCATGGACGTTCGT |
| CTGF-qPCR-R | AACGTGTCTTCCAGTCGGTAAG |
| ANKRD1-qPCR-F | GAAACAACGAGAGGCAGAGCTC |
| ANKRD1-qPCR-R | AGAAACGTAGGCACATCCACAG |
| HK1-qPCR-F | AGTGGAAGGAGCAGATGTGGTC |
| HK1-qPCR-R | TGTAGCAAGCATTGGTGCCAGTG |
| HK2-qPCR-F | CTAAACTAGACGAGAGTTTCCTG |
| HK2-qPCR-R | CTCACAGTTGTGGTCATCATAAC |
| G6PD-qPCR-F | CGAGGCCGTCACCAAGAAC |
| G6PD-qPCR-R | GTAGTGGTCGATGCGGTAGA |
| 6PGL-qPCR-F | GGAGCCTCGTCTCGATGCTA |
| 6PGL-qPCR-R | GAGAGAAGATGCGTCCGGT |
| 6PGD-qPCR-F | GTGGCCCCACATCAAGACC |
| 6PGD-qPCR-R | GTCCCCATACTCTATCCCGTT |
| RPI-qPCR-F | AGTGCTGGGAATTGGAAGTGG |
| RPI-qPCR-R | GGGAATACAGACGAGGTTCAGA |
| 18srRNA-F | GATCCATTGGAGGGCAAGTCT |
| 18srRNA-R | CCAAGATCCAACTACGAGCTTTT |
|  |  |
| sgRNA sequence |  |
| Name | 5'-3' |
| ISG15-KO | GGTACGGCTGACGCAGACCG |
| UbCH8-KO | GGCTTGAACGGATACTCCGG |
| HERC5-KO | GAGTTAATAATGATTGCTGG |
| 6PGL-KO | GTCTGGAGAGAAGATGCGTC |
| SMADs-KO1 | GGAGTGCGCTTATACTACAT |
| SMADs-KO2 | GCTACTGTCACTAAGGCATT |
| SMADs-KO3 | GAGACGACCATCAAGAGACC |
| TEADs-KO | GTGGCTGGAGACCTGCTTCC |
|  |  |
| Primers used for plasmid construction |  |
| Name | 5'-3' |
| YAP-WT-HA-F | GTACGGTACCATGGATCCCGGGCAGCAGCCGCCGC |
| YAP-WT-HA-R | GTACGATATCTCAAGCGTAGTCTGGGACGTCGTATGGGTATAACCATGTAAGAAAGCTTTCTTT |
| YAP-K280R-HA-F | CAGCCACCACCCCTGGCTCCCCAGA |
| YAP-K280R-HA-R | AGCCAGGGGTGGTGGCTGCCGCACTGGAGCACTCTGACTG |
| YAP-K321R-HA-F | CAGCAAGAACTGCTTCGGCAGGCAA |
| YAP-K321R-HA-R | AAGCAGTTCTTGCTGCCGCAGCCGCAGCCTCTCCTTCTCC |
| YAP-K497R-HA-F | GTACGGTACCATGGATCCCGGGCAGCAGCCGCCGC |
| YAP-K497R-HA-R | GTACGATATCTCAAGCGTAGTCTGGGACGTCGTATGGGTATAACCATGTAAGAAAGCTTTCCCG |
|  |  |
| Primers used for ChIP-qPCR |  |
| Name | 5'-3' |
| ChIP-P1-F | CCGGTTCAAGCGATTCTCCTGCCTC |
| ChIP-P1-R | GCGCGGTGGCTGACGCCTGTAAT |
| ChIP-P2-F | TCAGAAAATCCCTGCCACCTCGATAGG |
| ChIP-P2-R | ACATGCGCAGTGAGGCAAGTGAAG |
| ChIP-P3-F | AGCCACCGCGCCCGGCGGTGCAGGAATTTTAAAA |
| ChIP-P3-R | GGCAGGGATTTTCTGAAGGA |
| ChIP-P4-F | CTTACACACATCACTGAAATATCCCACTCTC |
| ChIP-P4-R | GCGAAAGCCACTGCGCCTGGCCATGCTT |
| ChIP-P5-F | GAGGCCGAGGCAGGAAGATCGCT |
| ChIP-P5-R | CAGTGAGCCGAGATCGTGCCACT |
| ChIP-P6-F | CCCCTTCCAGCCCTCTGTCCAGGCAAGAA |
| ChIP-P6-R | CTAGGCTGATCTTGAACCCCT |
| ChIP-P7-F | ACAAGCATATTTTCATCCCCTACT |
| ChIP-P7-R | CGCCCGGTTAATTTTTTGTGTTTTCAGTAGAG |
| ChIP-P8-F | ATTTTAAAACTCACCAAAATACGCCCTCC |
| ChIP-P8-R | GGCAGGGATTTTCTGAAGGA |
|  |  |
| Primers used for promoter analysis |  |
| Name | 5'-3' |
| 6PGL-Promoter-WT-F | GTACGGTACCAAATCCACTACCTGTGTGAGTTGAACTGCGC |
| 6PGL-Promoter-WT-R | CTAGGCTAGCTACTAGCGACGGCCGTAGGGAGCGCTTC |
| 6PGL-Promoter-MutP1-F | CGCGCCCGGCGGTGCAGGAATTTTA |
| 6PGL-Promoter-MutP1-R | GCACCGCCGGGCGCGGTAATCCCAACACTTTGGGAGGCCG |
| 6PGL-Promoter-MutP2-F | CGATAGGACGTCCTCTTCTTGAGCT |
| 6PGL-Promoter-MutP2-R | GAGGACGTCCTATCGTTTCTGAAGGAGGGCGTATTTTGGT |
| 6PGL-Promoter-MutP3-F | AAACTCACCAAAATACGCCCTCCTT |
| 6PGL-Promoter-MutP3-R | GTATTTTGGTGAGTTTACCGCCGGGCGCGGTGGCTGACGCC |
|  |  |
| Probes used for EMSA |  |
| Name | 5'-3' |
| EMSA-P1-F | GGATTACAGGCGTCAGCCACCGCGCC |
| EMSA-P1-R | GGCGCGGTGGCTGACGCCTGTAATCC |
| EMSA-P2-F | TCAGAAAATCCCTGCCACCTCGATAG |
| EMSA-P2-R | CTATCGAGGTGGCAGGGATTTTCTGA |
| EMSA-P3-F | CGGCGGTGCAGGAATTTTAAAACTC |
| EMSA-P3-R | GAGTTTTAAAATTCCTGCACCGCCG |
| EMSA-P4-F | TAGCTTAAAAAATTCAACAAGCATG |
| EMSA-P4-R | CATGCTTGTTGAATTTTTTAAGCTA |
